# Pediatric size phlebotomy tubes and transfusions in adult critically ill patients: a pilot randomized controlled trial

**DOI:** 10.1186/s40814-020-00657-3

**Published:** 2020-08-08

**Authors:** Javier Barreda Garcia, Jonathan Z. Xian, Claudia Pedroza, Moiz Salahuddin, Garbo Mak, Anabelle Keene, Sujith V. Cherian, Alisha Y. Young, Praveen Vijhani, Pratik B. Doshi

**Affiliations:** 1 Division of Critical Care Medicine, Department of Internal Medicine, The University of Texas Health Science Center at Houston, 6431 Fannin Street, MSB 1.434, Houston, TX 77030 USA; 2 Center for Clinical Research and Evidence-Based Medicine, The University of Texas Health Science Center at Houston, Houston, TX USA; 3 Memorial Hermann Hospital Texas Medical Center, Houston, TX USA; 4 Baptist Health Hospital, Corbin, KY USA

**Keywords:** Blood transfusion, Phlebotomy, Critical care, Clinical trial

## Abstract

**Background:**

Transfusion of red blood cells (RBC) is common, can have adverse effects, and is a costly and limited resource. Interventions that reduce iatrogenic blood losses could reduce transfusions. The objectives of this pilot trial were to assess the feasibility (acceptability of the intervention and suitability of eligibility criteria) and potential effectiveness of pediatric size phlebotomy tubes in adult critically ill patients.

**Methods:**

We conducted a pilot, randomized controlled trial in the medical intensive care unit (ICU) of a university-affiliated, tertiary care referral hospital from November 2017 to September 2018. A total of 200 patients with hemoglobin of at least 7 g/dL and without bleeding were randomized to pediatric or adult size phlebotomy tubes. Stratification was according to baseline hemoglobin (7–9.49 g/dL, 9.5–11.99 g/dL, and 12 g/dL or greater). Acceptability was measured via the number of blood test recollections and the number of patients that discontinued the use of pediatric tubes. The suitability of patient eligibility criteria was determined by identifying baseline characteristics associated with RBC transfusions. Potential effectiveness was estimated from the time to RBC transfusion or to hemoglobin level below 7 g/dL.

**Results:**

The use of pediatric tubes was acceptable as patients experienced a low number of tests recollections (on average 1 every 57 days), and none of the participants discontinued their use. The baseline hemoglobin category was the only factor that appeared to be independently associated with RBC transfusions. A total of 6 patients (6%) in the pediatric tube group and 11 patients (11%) in the adult tube group (hazard ratio, 0.69; 95% CI, 0.25 to 1.9) received an RBC transfusion or reached hemoglobin below 7 g/dL. Almost all of these patients (16 of 17 participants) had baseline hemoglobin of 7–9.49 g/dL.

**Conclusions:**

This pilot study suggests that pediatric phlebotomy tubes are acceptable to patients and can therefore be used in adult ICU patients. A future study should focus on patients with hemoglobin levels below 9.5 g/dL, as these patients have a high risk of transfusions. This intervention has the potential of being successful in selected patients. A definitive trial is warranted.

**Trial registration:**

ClinicalTrials.gov, NCT03286465. Retrospectively registered on September 18, 2017.

## Background

Red blood cell (RBC) transfusions are common in critically ill patients [1, 2]. An intervention that reduces RBC transfusions could help save this limited and costly resource and prevent complications associated with its use [3]. The volume of diagnostic phlebotomies has been associated with anemia [4, 5] and RBC transfusions [6, 7] in hospitalized patients. Therefore, a reduction in phlebotomy blood losses could potentially prevent blood transfusions.

The use of pediatric size phlebotomy tubes in adults has been proposed to reduce RBC transfusions [8]. Adult phlebotomy volumes exceed pediatric ones for the same tests [9]. However, it has not been proven that they can prevent transfusions or improve clinical outcomes. A retrospective study showed no major difference between the admission and the discharge hemoglobin of patients admitted to a general internal medicine floor before and after the introduction of pediatric basic metabolic panel tubes [10]. The only randomized controlled trial (RCT) that has used pediatric tubes in adults included 49 patients in the intensive care unit (ICU) and suggested a reduction in the decline in hemoglobin in the group using pediatric tubes [11]. Only 5 patients in the trial required a transfusion, which restricted the analysis of this outcome. Further limiting the widespread adoption of pediatric tubes in adults is the longer time required for their collection and laboratory processing compared with adult tubes.

Before proceeding with an RCT that evaluates whether pediatric tubes reduce RBC transfusions in adults, several knowledge gaps need to be addressed. These include the acceptability of pediatric tubes in the adult ICU setting, the best inclusion criteria, and the likelihood of success. We performed a pilot RCT in medical ICU patients to investigate these questions.

## Methods

### Design

From November 2017 through September 2018, we conducted an unblinded RCT, in which pediatric size phlebotomy tubes were compared with adult size tubes among critically ill patients admitted to the medical ICU of a university-affiliated, level 1 trauma, tertiary care referral center. The trial was approved by the institutional review board at the University of Texas Health Science Center at Houston with a waiver of informed consent. The main basis of this waiver was that the intervention involved no more than minimal risk to the participants. These small size phlebotomy tubes were already routinely used in the pediatric ICU and in selected patients in our adult medical ICU. The trial was registered at ClinicalTrials.gov (NCT03286465) before initiation. There was no funding source for this study. All authors vouch for the accuracy and for the fidelity of the trial to the protocol.

### Patients

All adults (18 years of age or older) admitted to the medical ICU were screened for eligibility. Inclusion criteria were hemoglobin of at least 7 g/dL and randomization expected within 12 h of ICU admission. Key exclusion criteria were bleeding and surgical admission diagnosis (for details see Additional file 1).

### Randomization

The random allocation sequence was generated by a statistician, and randomization was implemented in REDCap. Patients were randomized in permuted blocks of 4 or 6 and a 1 to 1 ratio, into one of two groups: the pediatric tube group or the adult tube group. Randomization was stratified according to the most recent hemoglobin (7 to 9.49 g/dL, 9.5 to 11.99 g/dL, and 12 g/dL or greater).

### Interventions

A pre-intervention phase was implemented to standardize the pediatric tube phlebotomy process (Additional file 1: Supplementary Methods). Following randomization, hematology, chemistry, and coagulation tests were collected using pediatric or adult tubes according to the intervention group (Additional file 1: Table S1). Reference tables with recommended phlebotomy volumes for individual and multiple tests were placed near the rooms of patients in the pediatric tubes group (Additional file 1: Tables S2 and S3). In the pediatric tube group, 2 tubes were sent whenever chemistry testing required more than a basic metabolic panel. Blood cultures and immunology tests were collected in adult size tubes in both groups as recommended by hospital laboratory staff to assure adequate processing. Arterial blood gasses and lactate levels were also collected in the same tubes in both groups as only a single size tube was available in the hospital for each of these tests. The phlebotomy process in the adult tube group followed usual practice. The indications for RBC transfusions were at the discretion of the treating physicians.

The intervention period started at randomization and ended when the patient received an RBC transfusion, the hemoglobin was less than 7 g/dL, the patient was discharged from the ICU or died, clinical bleeding or surgery occurred, comfort care measures were initiated, or the patient stayed in the ICU for more than 30 consecutive days. Diagnostic phlebotomies followed regular practice after any of these conditions occurred.

### Data collection

We collected baseline information that included demographic characteristics, comorbidities, and ICU admission features. The patient’s gender, height, and weight were used to estimate their total blood volume based on Nadler’s equation [12]. After randomization, we obtained data regarding factors influencing blood loss and anemia. Phlebotomy volumes were estimated based on the number of blood tests and the maximum volume of each tube (Additional file 1: Table S1). We recorded the baseline hemoglobin and the first hemoglobin available every day during the intervention period.

### Outcomes

The outcomes that assessed the acceptability of pediatric tubes were the proportion of patients that discontinued their use and the number of test recollections (average per patient per day and proportion in the group with blood redraws). We did not specify a priori criteria for success regarding these outcomes. These were evaluated by interviewing the nursing staff. Any sample requiring recollection was considered inadequate. This outcome was not recorded in the adult tube group as we thought that asking the nurses if a blood recollection had occurred could mislead them to use pediatric tubes.

The suitability of patient eligibility criteria was evaluated by assessing the association between several baseline characteristics and the time to RBC transfusion or hemoglobin less than 7 g/dL. The goal was to identify inclusion criteria that would help select patients at high risk of transfusions. The variables included were age, the Acute Physiology and Chronic Health Evaluation (APACHE) II score, the Charlson comorbidity index, end-stage renal disease, the baseline hemoglobin category, and the estimated blood volume of each patient. A variable was considered suitable to be used as eligibility criteria if it was independently associated with the time to RBC transfusion or hemoglobin less than 7 g/dL in the multivariable analysis.

The primary outcome to assess the potential effectiveness of this intervention was the time from randomization to RBC transfusion or hemoglobin less than 7 g/dL (for details see Additional file 1: Supplementary Methods). Hemoglobin below 7 g/dL was included as part of this outcome as it is the most common hemoglobin threshold for transfusion [13] and to minimize bias related to the treating physicians knowing the group assignments. Other outcomes to assess the potential success of pediatric tubes were the rate of change in hemoglobin, ICU length of stay, and ICU mortality. The goal was to estimate the potential success of this intervention. However, this study was not designed to reach definitive conclusions regarding effectiveness. We did not specify a threshold of potential effectiveness to determine if a definitive trial was warranted.

We also evaluated the number of blood tests, phlebotomy volumes, and adverse events related to RBC transfusions. We did not specify any criteria to decide whether to proceed with a future trial.

### Statistical analysis

As this was a pilot study, no sample size calculation was performed. We planned to randomize a total of 200 patients (100 in each group). This number of participants was chosen because it was thought it would be large enough to estimate the acceptability of pediatric tubes, suitability of eligibility criteria, and potential effectiveness, while allowing completion of the study in a reasonable time frame.

All analyses were conducted according to the intention-to-treat principle. The Cox proportional hazard model was used to assess the association between the baseline characteristics listed in the “Outcomes” section and the time to RBC transfusion. The intervention groups were compared in regard to the time to RBC transfusion or hemoglobin less than 7 g/dL using a Cox proportional hazard model stratified according to ICU admission hemoglobin group. We report the hazard ratio and 95% CI. Censoring events were death, ICU discharge, clinical bleeding, surgery, initiation of comfort care measures, and end of the study. This outcome was also characterized using Kaplan-Meier estimates and the log-rank test.

The change in hemoglobin was assessed using a multilevel mixed model that included intervention group, time, and admission hemoglobin group as fixed effects and random effects (participants and duration of the intervention). We used a quadratic term for time to capture the non-linear change while accounting for within-subject correlation.

Additional binary outcomes were compared between treatment groups using log binomial models. The days in the ICU, number of blood tests, and phlebotomy volumes were compared between interventions using negative binomial models. All of these analyses were adjusted for our stratification variable, the admission hemoglobin group. A post hoc analysis was conducted using an unadjusted, log-binomial Bayesian model to estimate the probability of pediatric tubes reducing the rate of transfusion in the subgroup of patients with lowest admission hemoglobin. We used a prior distribution centered on a relative risk of 0.69 with 95% credible interval of 0.13 to 3.8, based on the only RCT that has used pediatric tubes in adults [11].

All analyses were performed with the statistical software R, version 3.5.2, or STATA, version 15.1. Our results are presented with confidence intervals but not *p* values, as pilot studies are not powered to look at effectiveness.

## Results

### Baseline characteristics and flow of the participants

**Fig. 1:**
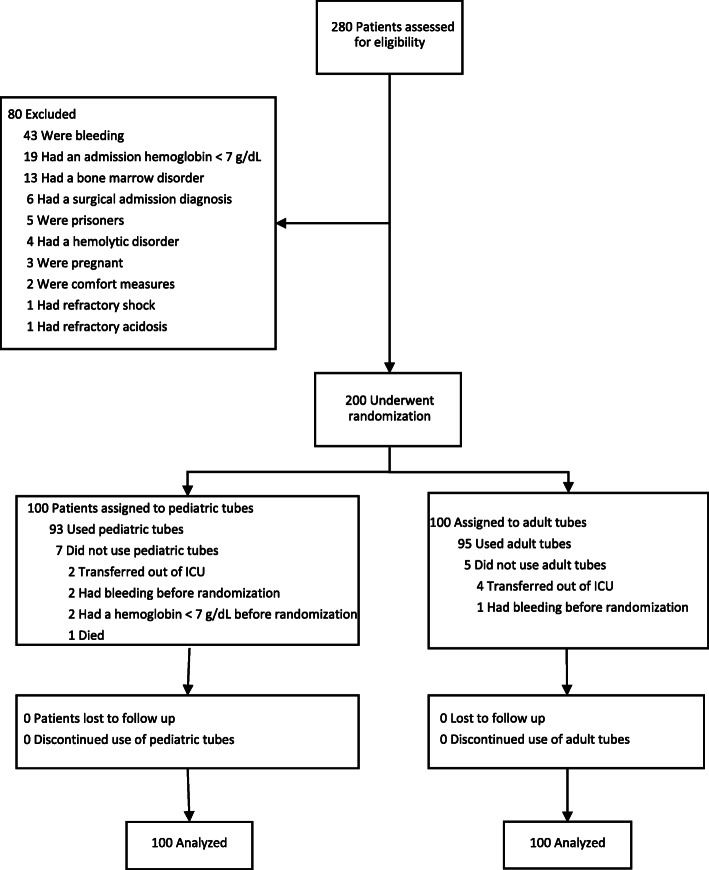
Study flow diagram

We screened 280 patients for the study. A total of 200 patients underwent randomization (Fig. 1). The two groups were similar in regard to most of their baseline characteristics (Table 1). A greater percentage of patients in the pediatric tube group had an admission diagnosis related to infection (55% vs. 46%), acute renal failure (40% vs. 31%), or a non-infectious inflammatory disorder (19% vs. 11%).

**Table 1:** Participant characteristics at baseline

Characteristic	Pediatric tubes (*n* = 100)	Adult tubes (*n* = 100)
Age, year, mean (SD)	55 (16.7)	57 (18.5)
Women, *n* (%)	45 (45)	51 (51)
Race, *n* (%)		
White	38 (38)	39 (39)
Black	36 (36)	46 (46)
Other	26 (26)	15 (15)
ICU admission hemoglobin, g/dL		
Median (IQR)	11.4 (9.3–12.7)	11.3 (9.2–12.7)
Mean (SD)	11.4 (2.4)	11.1 (2.3)
ICU admission hemoglobin category, *n* (%)		
7–9.49 g/dL	27 (27)	29 (29)
9.5–11.99 g/dL	32 (32)	32 (32)
≥ 12 g/dL	41 (41)	39 (39)
Time from ICU admission to randomization, h, median (IQR)	5.3 (1.5–8)	5.7 (2.6–8.6)
Estimated blood volume per participant, L, median (IQR)^a^	4.9 (4.2–5.6)	4.6 (3.9–5.4)
Chronic hemodialysis, *n* (%)	12 (12)	12 (12)
Chronic kidney disease but no dialysis, *n* (%)	13 (13)	11 (11)
Active malignancy, *n* (%)	5 (5)	5 (5)
Recent history of anemia, *n* (%)^b^	45 (45)	47 (47)
Charlson comorbidity index, median (IQR)^c^	3.0 (2–6)	4.0 (2–5)
ICU admission diagnosis, *n* (%)^d^		
Infection	55 (55)	46 (46)
Acute cardiovascular disorder	21 (21)	24 (24)
Acute renal failure	40 (40)	31 (31)
Metabolic disorder	32 (32)	36 (36)
Non-infectious inflammatory disorder^e^	19 (19)	11 (11)
Other	22 (22)	31 (31)
Invasive mechanical ventilation, *n* (%)	50 (50)	51 (51)
APACHE II score, median (IQR)^f^	22.0 (14-29)	23.0 (16-28)

*SD* standard deviation, *ICU* intensive care unit, *IQR* interquartile range, *APACHE* Acute Physiology and Chronic Health Evaluation

^a^The blood volumes were estimated according to Nadler method, which is based on the participant’s gender, height, and weight

^b^A recent history of anemia was defined as hemoglobin below 13 g/dL in men or 11.6 g/dL in women in the preceding 6 months

^c^The Charlson comorbidity index measures the effect of coexisting conditions on mortality, with scores ranging from 0 to 33. Higher scores indicate greater burden of illness

^d^The diagnosis are not mutually exclusive

^e^Non-infectious inflammatory disorders included conditions such as acute pancreatitis, asthma exacerbation, and chronic obstructive pulmonary disease exacerbation

^f^APACHE II scores are assessed on a scale from 0 to 71. Higher scores indicate higher risk of death

There were no patients in the adult tube group and 9 patients (9%) in the pediatric tube group that experienced at least one intervention crossover. Among those with intervention crossovers, the median number of occurrences per patient was 1 (IQR, 1 to 1).

### Potential factors influencing blood loss and anemia

There were no important differences in the number of the different types of blood tests between the pediatric and the adult tube groups (Table 2). The median percentages of the total phlebotomy volume accounted by different tests were as follows: hematology 9% (interquartile range [IQR], 3 to 18%), chemistries 21% (IQR, 11 to 32%), coagulation 0% (IQR, 0 to 5%), immunology 0% (IQR, 0 to 13%), lactate 0% (IQR, 0 to 11%), blood gasses 3% (IQR, 0 to 9%), and blood cultures 0% (IQR, 0 to 0%). The estimated phlebotomy volumes in the pediatric tube group were less than in the adult tube group (Table 2).

**Table 2:** Factors influencing blood loss and anemia during the intervention period

Procedure	Pediatric tubes (*n* = 100)	Adult tubes (*n* = 100)	Absolute difference (95% CI)	Risk ratio (95% CI)
Use of an arterial line, *n* (%)	25 (25)	17 (17)	8 (− 4 to 20)	1.5 (0.9 to 2.6)
Use of a central venous catheter, femoral vein catheter, or peripherally inserted central catheter, *n* (%)	31 (31)	21 (21)	10 (− 3 to 23)	1.5 (0.9 to 2.5)
Anticoagulation or anti-platelet agents, *n* (%)	38 (38)	35 (35)	3 (− 11 to 17)	1.1 (0.8 to 1.6)
Renal replacement therapy, *n* (%)	17 (17)	18 (18)	− 1 (− 12 to 10)	1 (0.5 to 1.8)
Total number of blood tests per patient, median (IQR)^a^				
Hematology	2.0 (1–3)	2.0 (1–4)	0	0.8 (0.6 to 1.1)
Chemistry^b^	5.0 (3–8)	4.0 (2–8)	NA	NA
Immunology	0 (0–1)	1.0 (0–1)	− 1	0.9 (0.7 to 1.4)
Coagulation	0 (0–1)	0 (0–1)	0	0.9 (0.4 to 1.8)
Lactate	1 (0–2)	0 (0–2)	1	1.1 (0.6 to 1.8)
Blood gasses	1 (0–3)	1 (0–4)	0	0.8 (0.5 to 1.3)
Blood culture bottles	0 (0–0)	0 (0–0)	0	1 (0.5 to 1.9)
Phlebotomy volume^c^				
Per participant per day, mL, median (IQR)	8.6 (4–18)	21.6 (15–31)	− 13	0.5 (0.4 to 0.6)
Total per participant, mL, median (IQR)	21 (7–38)	50 (27–100)	− 29	0.4 (0.3 to 0.6)
Total per participant as a percentage of their estimated blood volume, %, median (IQR)	0.4 (0.2–0.7)	1.1 (0.6–2.1)	− 0.7	0.3 (0.2 to 0.5)

*CI* confidence interval, *IQR* interquartile range, *NA* not applicable

^a^The mean (range) of the total number of tests per patient, pediatric tubes vs adult tubes, were as follows: hematology 2.7 (0–19) vs 3.2 (0–18), chemistry 8.1 (0–52) vs 6.1 (0–34), immunology 0.7 (0–5) vs 0.7 (0–5), coagulation 1.3 (0–16) vs 1.5 (0–31), lactate 1.8 (0–32) vs 1.6 (0–25), blood gasses 3.2 (0–61) vs 3.6 (0–30), and blood cultures 0.5 (0–6) vs 0.5 (0–4)

^b^In the pediatric tube group, the chemistry number is the number of tubes sent for testing. In this group, there was variable use of 1 or 2 tubes according to the type of chemistry test performed (as specified by the laboratory). Therefore, a comparison with the adult tubes group in regards to the number of chemistry tests was not possible

^c^The maximum volume of each tube was used in this calculation. In the pediatric tube group, the volumes were 0.5 mL for hematology, 0.6 mL for chemistries, and 2.5 mL for coagulation tests. In the adult tube group, the volumes were 5.5 mL for hematology, 5 mL for chemistries, and 3.8 mL for coagulation. Tube volumes for lactate, immunology, arterial blood gases, and blood cultures were 5 mL, 6.5 mL, 1 mL, and 10 mL, respectively

Compared to adult tube group, patients in the pediatric tube group had higher rates of use of arterial lines (17% vs 25%) and central venous catheters (21% vs 31%). The two groups were comparable in the use of medications associated with blood loss and dialysis (Table 2).

### Outcomes evaluating acceptability

There were no patients that discontinued the use of pediatric phlebotomy tubes. The median and the mean number of test recollections per patient per day in the pediatric tubes group were 0 (IQR, 0 to 0) and 0.0176 (standard deviation, 0.08), respectively, representing an average of 1 per patient every 57 days. A total of 7 patients (7%) had at least one and none had more than one phlebotomy recollections in the pediatric tubes group.

### Suitability of eligibility criteria

The baseline hemoglobin category was the only factor that appeared to be independently associated with RBC transfusions (Additional file 1: Supplementary Figure). Patients with a baseline hemoglobin of 7–9.49 g/dL accounted for almost all the patients that met criteria for an RBC transfusion: 16 patients (28%) in the lower hemoglobin group (7–9.49 g/dL), 1 patient (1.6%) in the middle hemoglobin group (9.5–11.99 g/dL), and no patients in the higher hemoglobin group (12 g/dL or greater). From the post hoc Bayesian analysis, we estimated that there is an 82% probability that pediatric tubes reduce the relative risk of RBC transfusions by more than 25% in patients in the lower hemoglobin group.

### Outcomes evaluating potential effectiveness

**Fig 2:**
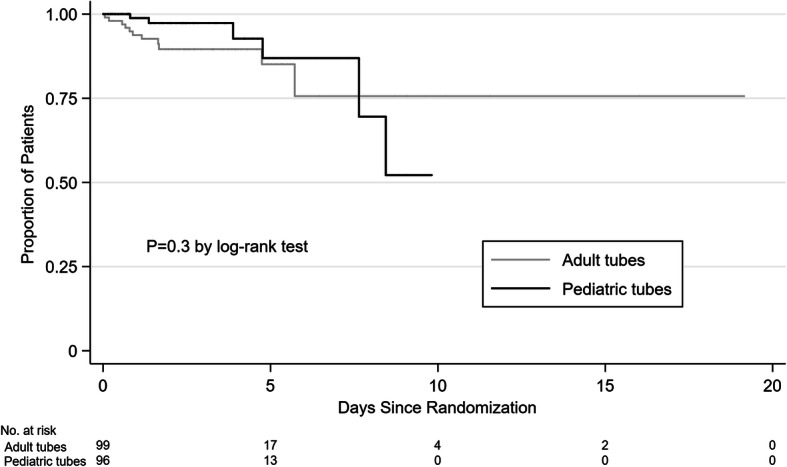
Time to transfusion or hemoglobin less than 7 g/dL by intervention group

A total of 6 patients (6%) in the pediatric tube group and 11 patients (11%) in the adult tube group received an RBC transfusion or reached hemoglobin of less than 7 g/dL (hazard ratio, 0.69; 95% CI, 0.25 to 1.9) (Table 3 and Fig. 2). All of these patients had hemoglobin of less than 7 g/dL, and 13 patients (76%) were transfused. In the pediatric tube group, the 2 patients that were not transfused increased their hemoglobin above 7 g/dL without intervention. In the adult tube group, the first patient that was not transfused was transitioned to hospice and the second patient died, both shortly after the low hemoglobin was identified. The absolute difference in this outcome was − 5% (95% CI, − 14 to 4%), in favor of the pediatric tube group. A total of 1 patient in the pediatric tube group and 6 patients in the adult tube group met this outcome in the first 24 h after randomization. The median duration of the interventions was 2 days in both the pediatric tube group (IQR, 1.2 to 3.4) and the adult tube group (IQR, 1.4 to 3.9).

**Table 3:** Outcomes assessing potential effectiveness

Outcomes	Pediatric tubes (*n* = 100)	Adult tubes (*n* = 100)	Absolute difference (95% CI)	Hazard or risk ratio(95% CI)
RBC transfusion or hemoglobin <7 g/dL, n (%)	6 (6)	11 (11)	− 5 (− 14 to 4)	0.69 (0.25 to 1.9)
By baseline hemoglobin				
7 to 9.49 g/dL	5 (18.5)	11 (38)	− 19 (− 42 to 3.5)	0.57 (0.2 to 1.67)
9.5 to 11.99 g/dL	1 (3)	0	3 (− 3 to 9)	NA
≥ 12 g/dL	0	0	NA	NA
Rate of change in hemoglobin, g/dL/day	− 0.19	− 0.14	− 0.05 (− 0.15 to 0.06)	NA
By baseline hemoglobin				
7 to 9.49 g/dL	− 0.07	− 0.18	0.11 (− 0.1 to 0.3)	NA
9.5 to 11.99 g/dL	− 0.18	− 0.05	− 0.13 (− 0.3 to 0.01)	NA
≥ 12 g/dL	− 0.29	− 0.24	− 0.05 (− 0.3 to 0.2)	NA
Mortality in the ICU, *n* (%)	9 (9)	10 (10)	− 1 (− 10 to 8)	0.9 (0.4 to 2.1)
Days in the ICU, median (IQR)	2.5 (1.5–4)	2.5 (1.5–5)	0	0.9 (0.7 to 1.2)

*CI* confidence interval, *RBC* red blood cell, *NA* not applicable, *ICU* intensive care unit, *IQR* interquartile range

Overall, the hemoglobin level declined by 0.16 g/dL per day (95% CI, − 0.21 to − 0.10) in the study participants. The change in hemoglobin was similar in the two treatment groups (Table 3). There was no evidence of an association between the change in hemoglobin and any other variable. Mortality and length of stay were similar between the two groups (Table 3).

### Other outcomes

There were no reported adverse events related to RBC transfusions.

## Discussion

In our pilot study comparing pediatric and adult phlebotomy tubes, we found that pediatric tubes can be used in the adult ICU setting. A future study should focus on patients with hemoglobin levels below 9.5 g/dL. This intervention has good potential of being effective in reducing RBC transfusions in selected critically ill patients. These results provide support for a larger RCT.

Our findings suggest that the use of pediatric phlebotomy tubes is acceptable in an adult ICU. Potential barriers to the use of pediatric tubes include the possibility of inadequate blood samples and the additional steps that the nurses and the laboratory need to do for their processing (need to uncap the tubes during blood collection and analysis) compared with adult tubes. Participants using pediatric tubes had a low number of test recollections, and none of them had to stop their use. Considering the low patient to nurse ratios in the ICU, it is likely that their use will remain acceptable to the nursing staff in a larger trial. However, the ability of different laboratories to handle the extra work is expected to be variable.

This study helps define the inclusion criteria of a future trial and shows that pediatric phlebotomy tubes are a promising potential intervention to reduce RBC transfusions. Pediatric tubes were associated with a 31% reduction in the risk of RBC transfusions compared with adult tubes. However, this intervention might not benefit all patients alike. Our results suggest that patients with hemoglobin levels above 9.5 g/dL may be unlikely to benefit from pediatric tubes as only a minority of these patients needed a transfusion in our study. In contrast, patients with hemoglobin levels below 9.5 g/dL appear to be the best candidates to use pediatric tubes. This group of patients has a high risk of transfusions, and we estimate that pediatric tubes have a high probability of reducing them. This potential benefit seems worth exploring in a definitive RCT. Restricting this intervention to patients with low hemoglobin will have the added benefit of reducing the workload to the laboratory.

Our study supports the design of a definitive RCT. We suggest that a future trial also excludes patients with bleeding and allows transfusions to follow usual practice. A measure of RBC transfusions could also be a reasonable primary outcome. A few amendments to the study design are recommended: limiting participants to those with hemoglobin less than 9.5 g/dL (due to their higher risk of transfusions), testing in multiple ICU settings (to improve external validity), and continuing the intervention beyond the ICU (to allow longer exposure to the intervention).

This pilot trial has several strengths. We evaluated the acceptability of pediatric tubes and the suitability of eligibility criteria in a group of participants that is likely representative of a typical medical ICU population. The average age [14], APACHE II score [14, 15], use of mechanical ventilation [14], and ICU length of stay [14, 16, 17] of our participants are comparable to other critically ill patients. However, the ICU mortality is lower than in a large worldwide observational study (10% vs 16%) [14]. The percentage of patients in our control group that met criteria for an RBC transfusion resembles other non-bleeding ICU patients [11, 18]. The daily phlebotomy volume of the adult tube group is lower [2, 11, 18, 19], similar [4, 7, 20], or higher [6], than in previous studies in the ICU.

Our study has several limitations. The assessment of the number of test recollections was susceptible to bias as it was based on interviews. Furthermore, we did not record phlebotomy recollections in the adult tube group which did not allow us to do a comparison with the pediatric tubes group. However, the number of recollections in the pediatric tubes was consistent with previous studies [11, 20, 21]. Our estimates of potential effectiveness could have been affected by the lack of blinding. Our findings might not apply in the context of other ICU settings or transfusion triggers.

## Conclusions

This pilot RCT shows that pediatric phlebotomy tubes can be used in adult ICU patients. Patients with hemoglobin levels below 9.5 g/dL are at higher risk of transfusions and should therefore be the focus of a future trial. This intervention could reduce transfusions in selected patients. A multicenter trial to determine the effectiveness of this intervention is warranted.

## Supplementary information

**Additional file 1: **Supplementary Methods. **Table S1.** Phlebotomy tubes volume. **Table S2.** Draw Volume for Individual Tests in the Pediatrics Tubes Group. **Table S3.** Draw Volume for Multiple Tests in the Pediatrics Tubes Group. **Supplementary Figure.** Time to Transfusion or Hemoglobin Less than 7 g/dL by Hemoglobin Group.

## Data Availability

The dataset used during the current study is available from the corresponding author on reasonable request.

## Abbreviations

RBC: Red blood cells
RCT: Randomized controlled trial
ICU: Intensive care unit
CI: Confidence interval
APACHE: Acute Physiology and Chronic Health Evaluation
IQR: Interquartile range
