# Supplementary material for: Pediatric size phlebotomy tubes and transfusions in adult critically ill patients: a pilot randomized controlled trial
Source: Pilot Feasibility Stud. 2020 Aug 8;6:112. doi: 10.1186/s40814-020-00657-3 (PMC7414662; doi:10.1186/s40814-020-00657-3)
Supplement: Supplementary file 1 — Additional file 1: Supplementary Methods. Table S1. Phlebotomy tubes volume. Table S2. Draw Volume for Individual Tests in the Pediatrics Tubes Group. Table S3. Draw Volume for Multiple Tests in the Pediatrics Tubes Group. Supplementary Figure. Time to Transfusion or Hemoglobin Less than 7 g/dL by Hemoglobin Group. [file 40814_2020_657_MOESM1_ESM.docx]

**Supplemental Appendix**

**Pediatric Size Phlebotomy Tubes and Transfusions in Adult Critically Ill Patients: A Pilot Randomized Controlled Trial**

Javier Barreda Garcia, MD, Jonathan Z. Xian, MD, Claudia Pedroza, PhD, Moiz Salahuddin, MD, Garbo Mak, MD, Anabelle Keene, AGACNP-BC, Sujith V. Cherian, MD, Alisha Y. Young, MD, Praveen Vijhani, MD, Pratik B. Doshi, MD

**Corresponding Author:** Javier Barreda Garcia, MD

The University of Texas Health Science Center at Houston

6431 Fannin Street, MSB 1.434

Houston, TX 77030

**TABLE OF CONTENTS**

**Supplementary Methods** ………………………………………………………………………. 3

Inclusion and Exclusion Criteria ………………………………………………………... 3

Interventions …………………………………………………………………………….. 4

Assessment of the Time to RBC Transfusion or Hemoglobin Less than 7 g/dL ……...... 5

**Table S1.** Phlebotomy tubes volume …………………………………………........................... 6

**Table S2.** Draw Volume for Individual Tests in the Pediatrics Tubes Group ……………….… 7

**Table S3.** Draw Volume for Multiple Tests in the Pediatrics Tubes Group ………………….... 8

**Supplementary Figure.** Time to Transfusion or Hemoglobin Less than 7 g/dL by Hemoglobin Group ……………………………………………………………………………………….…… 9

\

**SUPPLEMENTARY METHODS**

**Inclusion Criteria**

Subjects were eligible for enrollment in the study if they met all of the following criteria:

1. Subjects 18 years of age or older.
2. New admission to the medical ICU.
3. ICU admission hemoglobin level of at least 7 g/dL. The ICU admission hemoglobin was the most recent hemoglobin available at the time of screening.
4. Randomization expected within 12 hours of admission to the ICU.

**Exclusion Criteria**

Subjects meeting any of the following criteria were not eligible for enrollment in the study:

1. Clinical bleeding. Defined as bleeding leading to a change in the frequency of hemoglobin monitoring or to an order for a medication, transfusion, procedure, or consultation intended to prevent or treat the blood loss.
2. Hemolytic disorder (e.g. sickle cell disease, hereditary spherocytosis, autoimmune hemolytic anemia).
3. Bone marrow disorder (e.g. aplastic anemia, marrow infiltration disorder, chemotherapy within the last 8 weeks).
4. Jehovah’s Witnesses.
5. Patient is comfort care measures only.
6. Refractory shock: Mean arterial blood pressure below 60 mmHg despite maximal doses of 3 vasopressors. Maximal doses were: Norepinephrine 70 mcg/min; vasopressin 0.04 units/min; epinephrine 35 mcg/min; dopamine 20 mcg/kg/min; phenylephrine 350 mcg/min.
7. Severe acidosis: pH below 7 in more than one arterial blood gas within 24 hours of ICU admission in the absence of diabetic ketoacidosis.
8. Surgical admission diagnosis.
9. Pregnancy.
10. Current prisoner.

**Interventions**

Pre-Intervention Phase

The study procedures were presented to the day and night ICU nurses one month prior to the beginning of the study to standardize the pediatric tubes phlebotomy process. Recommendations included the volume of blood to be drawn according to the type of test. Emphasis was placed in avoiding the collection of blood in excess of the necessary volume to run the tests.

Pediatric Tubes Group

Hematology, chemistry, and coagulation blood tests were collected using pediatric size tubes (**Table S1**). Two tubes were sent whenever chemistry testing required more than a basic metabolic panel (e.g. complete metabolic panel). Blood cultures and immunology tests were collected in adult size tubes in both groups as recommended by hospital laboratory staff to assure adequate processing. Arterial blood gases and lactate levels were also collected in the same tubes in both groups as only a single size tube was available in the hospital for each of these tests. If a test needed to be recollected due to an insufficient sample the repeat test was sent in an adult size tube. No more than 5 patients per day were participating in the pediatric tubes group as requested by the laboratory staff. Reference tables with recommended phlebotomy volumes for individual and multiple tests were placed near the rooms of patients in the pediatric tubes group (**Table S2 and S3**).

Patients assigned to the pediatric tubes group were identified as such by nursing staff by daily direct communication of the investigators with the day nurse, handoff between the day and night nurses, and signs outside the rooms and on the charts.

Adults Tubes Group

No recommendations were given regarding the phlebotomy process or phlebotomy volumes in the adult tubes group. All blood for testing was collected in adult size phlebotomy tubes (**Table S1**).

Both Groups

The frequency of blood testing and the indications for RBC transfusions were at the discretion of the treating physicians. The volume of fluid drawn to clear the intravenous lines could be returned to the patient in a sterile fashion in both groups whenever feasible.

**Assessment of the Time to RBC Transfusion or Hemoglobin Less than 7 g/dL**

The assessment of the primary outcome to evaluate the potential effectiveness of the intervention was done in three phases:

1. Initial evaluation. The outcome was initially assessed by two investigators that were blinded to the interventions. Outcomes that were scored as negative were final.
2. Review of positive outcomes. A third investigator, who was also blinded to the interventions, reviewed the outcomes that were considered positive. At this point, a positive outcome was final if there was concordance between the two reviews.
3. Review of discordant assessments. Any discordant result was resolved by consensus between all the outcome reviewers. The principal investigator, who was aware of the treatment assignments, also participated in the review of discordant evaluations.

| **Table S1** Phlebotomy Tubes Volume | | |
| --- | --- | --- |
| **Type of test** | **Adult tubes volume,^a^ mL** | **Pediatric tubes volume,^a^ mL** |
| Hematology | 4–5.5 | 0.25-0.5 |
| Chemistry (plasma) | 3-5 | 0.4-0.6 |
| Coagulation profile | 2.7-3.8 | 1.8-2.5 |
| Lactate | 2-5 | 2-5 |
| Immunology (serum) | 5-6.5 | 5-6.5 |
| Arterial blood gas | 1 | 1 |
| Blood cultures | 10 | 10 |
| ^a^Range is between the minimum volume to perform the test and the maximum tube volume. | | |

| **Table S2** Draw Volume for Single Tests in the Pediatrics Tubes Group | | |
| --- | --- | --- |
| **Test** | **Tube top color** | **Recommended volume to be sent to the lab, mL** |
| Complete blood count  or  Hemoglobin | Purple | 0.5 |
| Basic metabolic panel  or  Hepatic panel  or  Sodium  or  Potassium  or  Procalcitonin | Green | 0.6 |
| Complete metabolic panel  or  Basic metabolic panel + magnesium + phosphorus | Green | 1.2 (2 tubes) |
| Coagulation studies | Blue | 1.8 |
| Arterial blood gas, ionized calcium, lactate, immunology tests (e.g. viral hepatitis panel, rheumatologic tests), blood cultures | Same as adults | |

| **Table S3** Draw Volume for Multiple Tests in the Pediatrics Tubes Group | |
| --- | --- |
| **Tests** | **Recommended draw volume, mL** |
| CBC + CMP + procalcitonin + coagulation | 4 |
| CBC + CMP + coagulation | 3.5 |
| CBC + CMP  or  CBC + BMP + magnesium + phosphorus | 2 |
| CMP  or  BMP + magnesium + phosphorus | 1.2 |
| CBC + BMP | 1 |
| *CBC* complete blood count, *CMP* complete metabolic panel, *BMP* basic metabolic panel | |

**Supplementary Figure** Time to Transfusion or Hemoglobin Less than 7 g/dL by Baseline Hemoglobin Group


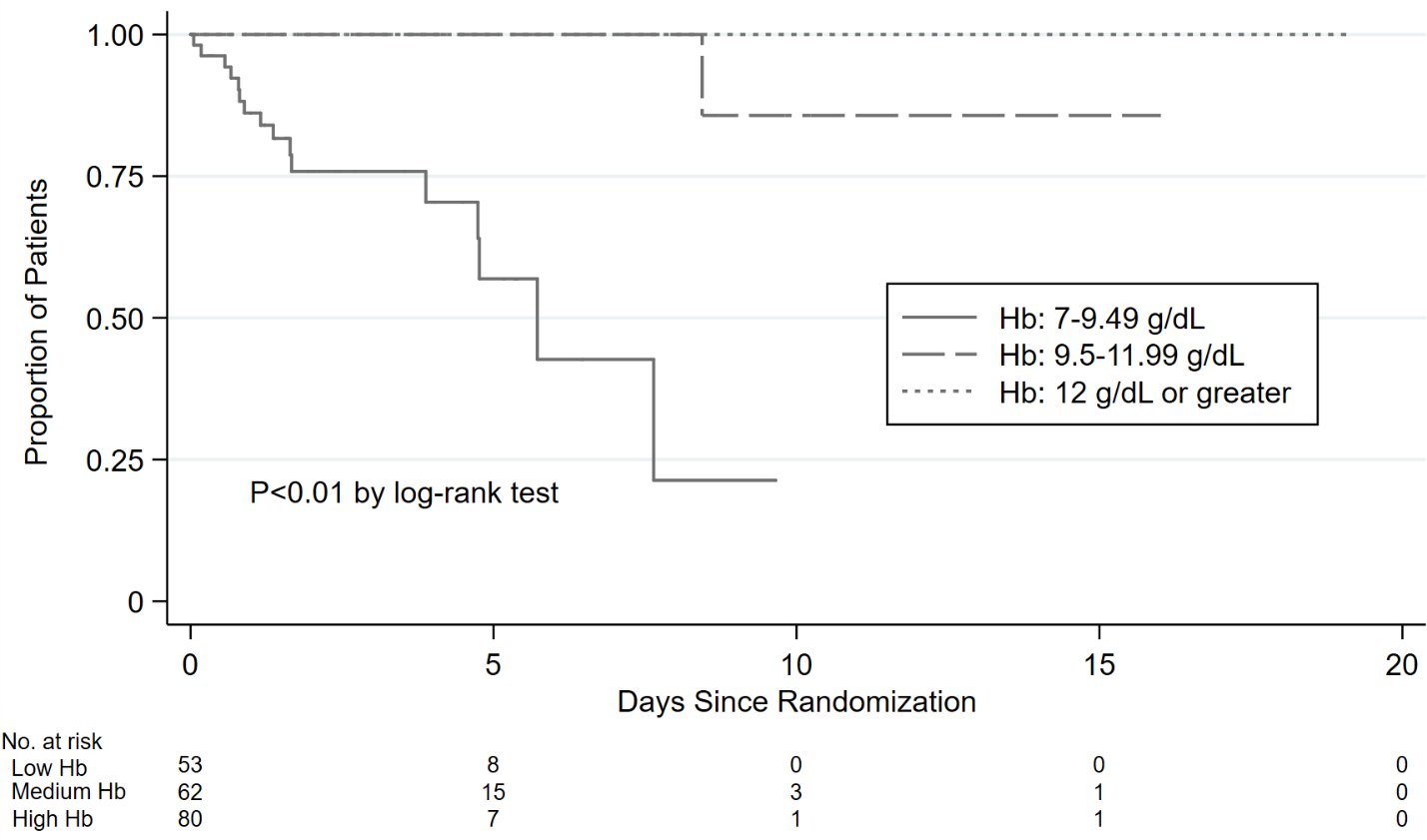


Hb indicates hemoglobin
